# Significance of NETs Formation in COVID-19

**DOI:** 10.3390/cells10010151

**Published:** 2021-01-14

**Authors:** Karolina Janiuk, Ewa Jabłońska, Marzena Garley

**Affiliations:** Department of Immunology, Medical University of Bialystok, J. Waszyngtona 15A, 15-269 Bialystok, Poland; karolinajaniukk@gmail.com (K.J.); ewa.jablonska@umb.edu.pl (E.J.)

**Keywords:** COVID-19, SARS-CoV-1, NETs, neutrophils

## Abstract

Severe contagious respiratory disease—COVID-19—caused by the SARS-CoV-2 coronavirus, can lead to fatal respiratory failure associated with an excessive inflammatory response. Infiltration and spread of SARS-CoV-2 are based on the interaction between the virus’ structural protein S and the cell’s receptor–angiotensin-converting enzyme 2 (ACE2), with the simultaneous involvement of human trans-membrane protease, serine 2 (TMPRSS2). Many scientific reports stress the importance of elevated recruitment and activity of neutrophils, which can form extracellular neutrophil traps (NETs) playing a significant role in the mechanism of combating pathogens, in the pathogenesis of COVID-19. Excessive generation of NETs during prolonged periods of inflammation predisposes for the occurrence of undesirable reactions including thromboembolic complications and damage to surrounding tissues and organs. Within the present manuscript, we draw attention to the impact of NET generation on the severe course of COVID-19 in patients with concurrent cardiovascular and metabolic diseases. Additionally, we indicate the necessity to explore not only the cellular but also the molecular bases of COVID-19 pathogenesis, which may aid the development of dedicated therapies meant to improve chances for the successful treatment of patients. We also present new directions of research into medications that display NETs formation regulatory properties as potential significant therapeutic strategies in the progress of COVID-19.

## 1. Introduction

The final month of 2019 became a permanent fixture in the history of the city of Wuhan. This occurred through the beginning of a global tragedy—the spreading pandemic of the SARS-CoV-2 virus. The virus-caused disease dubbed COVID-19 has been defined by the World Health Organization (WHO) as an acute infectious disease of the respiratory system, and because of a lack of a vaccine and tried treatment strategies, the SARS-CoV-2 virus has become an unprecedented challenge for the world’s health care systems [1]. There is, therefore, an urgent need to discover the pathomechanisms that drive this disease, which in turn, will allow the identification of potential therapeutic objectives.

For a significant portion of the population, the clinical course of a SARS-CoV-2 infection is asymptomatic or mildly symptomatic (which has been proclaimed as the driving force behind the pandemic). In these instances, the patients experience mild flu-like symptoms such as fever, cough, fatigue, weakness, and the loss of the sense of smell and/or taste [1,2].

However, for approximately 15% of patients, the infection leads to fatal respiratory failure resembling acute respiratory distress syndrome (ARDS) that is caused by a cytokine storm and/or multi organ dysfunction [3,4]. An important correlation between the severity of the progress of COVID-19 and age, being a male, and the concurrence of other diseases, such as diabetes, obesity, hypertension, chronic obstructive pulmonary disease, or cardiovascular diseases, has been observed [5].

## 2. Immunopathogenesis of COVID-19

By binding to angiotensin-converting enzyme 2 (ACE2), the SARS-CoV-2 virus can enter epithelial and/or endothelial cells, causing the simultaneous reduction in ACE2 expression within the tissue [6,7]. Sodhi et al. have been able to show that the weakened activity of ACE2 in the lungs of mice leads to the activation of des-Arg9 bradykinin (DABK)/bradykinin B1 Receptor (BKB1R) axis, the release of pro-inflammatory chemokines or the C-X-C motif chemokine 5 (CXCL5), CXCL1, macrophage inflammatory protein 2 (MIP2), and tumor necrosis factor (TNF-α) [8]. The result of these reactions ensuing from the dynamic variability in the expression of pulmonary ACE2 through the control of neutrophil recruitment is a significant factor coordinating the progression of inflammation [7]. SARS-CoV-2 utilizes the cell’s trans-membrane protease, serine 2 enzyme (TMPRSS2) to stimulate the structural protein S [9]. The level of expression and the location of ACE2 and TMPRSS2 within different tissue, therefore, determine the route through which SARS-CoV-2 spreads throughout the organism [5,10].

Systemic viral infections are usually accompanied by lymphocytosis, which is the effect of the increase in the pool of T CD8+ lymphocytes specific for the antigen. However, when it comes to COVID-19, a reduction in the number of lymphocytes along with neutrophilia is observed [11]. Increased production of transforming growth factor β (TGF-β), which has strong immunosuppressive activity, may be the cause of lymphopenia. An interesting observation concerning a SARS-CoV papain-like protein, which strongly triggered the ROS/p38 MAPK/STAT3 signaling pathway leading to the activation of the TGF-β1 promoter in the epithelium cells of the lungs has been made [5]. The increased neutrophil to lymphocyte ratio (NLR) as well as the reduced number of T CD8+ are described as an independent prognostication factor within the early phase of SARS-CoV-2 infection, signaling a severe progression of COVID-19 [5,11,12]. This is confirmed by research completed by Leppkes et al. (2020), where a significant rise in the overall number of leukocytes and neutrophilia and an elevated NLR in the severe progression of the disease, in comparison to its mild form as well as in patients who have recovered from the illness, were seen [13,14].

The main element in the pathogenesis of severe COVID-19 is the excessive production of cytokines (so-called “cytokine storm”) and its consequences [3,15,16,17]. At the site of infection within the respiratory tract, the pathogens are recognized by specialized alveolar epithelial cells (AEC), mast cells, and mononuclear phagocyte system (MPS) cells including monocytes, macrophages, and myeloid dendritic cells equipped with pattern recognition receptors (PRRs). Virus-related pathogen-associated molecular patterns (PAMPs) may trigger the release of a specific combination of PRRs and adaptor cells, allowing a particular immunologic response [5,18].

The replication of viruses characterized by the cytopathogenic effect such as coronaviruses causes numerous changes in a cell’s homeostasis leading to its death. Virus-induced cell death results in the release of damage-associated molecular patterns (DAMPs), which initiate the expression of classic PRRs in neighboring epithelial and endothelial cells. This is leading to the generation of additional pro-inflammatory cytokines and chemokines such as IL-6, interferon γ-induced protein 10 (IP-10), or macrophage inflammatory protein 1α (MIP1α) [19,20]. The cytokines recruit more effector cells intensifying the inflammatory process. IL-1β is produced by resident macrophages after the activation of pattern recognition receptors, which detect pathogen-associated molecular patterns or damage-associated molecular patterns. The most significant pulmonary macrophage PRRs are the NOD-, LRR-, and pyrin domain-containing protein 3 (NLRP3) receptor (a NOD3-like receptor), whose transcription is induced after the recognition of a pathogen’s structure. The NLRP3 recruits the apoptosis-associated speck-like protein (ASC) and pro-caspase 1, creating the NLRP3 inflammasome. The NLRP3 is a multimeric protein complex having the ability to activate the effective forms of pro-inflammatory cytokines including pro-IL-1β and pro-IL-18 that are then released outside of the cell. The expression of NLRP3 may also be induced by endogenous particles such as TNF-α or IL-1β through the activation of the TLR4-NF-κB pathway. There exists a biofeedback mechanism for the production of IL-1β, which can activate the NLRP3 inflammasome and inversely, creating a potential mechanism for the generation of excessive cytokine response. Additionally, IL-1β also induces the expression of several other cytokines such as TNF-α, IL-6, and IL-17 as well as other mediators of inflammation like inducible nitric oxide synthase (iNOS). It has been shown that IL-6, IL-8, IL-18, and MCP3 concentrations allowed differentiation between the severe and mild progression of the disease [13,21,22,23]. Wang et al. (2020) pointed out IL-6 as an early indicator of cytokine release syndrome occurring in patients suffering from COVID-19 [24]. Increased levels of IL-6 enable us to predict the possibility of respiratory failure. IL-6 concentrations are increased 2.9-fold in patients with complicated COVID-19 vs. uncomplicated. Additionally, neutrophilia and increased levels of IL-8 were found in the blood of severe cases of COVID-19 patients and were associated with poor disease prognosis [25,26,27]. Significantly elevated concentrations of C-reactive protein (CRP) produced as a result of macrophage stimulation by IL-1β, IL-6, or TNF-α have also been observed in COVID-19 patients [13].

## 3. Neutrophil Extracellular Traps

Neutrophils play a key role in the body’s innate immunological response constituting the first line of defense in the fight of a wide range of pathogens. During an infection, these cells’ protective duty is performed through phagocytosis, degranulation of antibacterial proteins, generation of reactive oxygen species (ROS), and the recruitment and activation of other immunocompetent cells. In 2004, Brinkmann et al. published a groundbreaking discovery concerning a significant property of neutrophilic granulocytes—their ability to generate extracellular neutrophil traps (NETs). The authors of this pioneering article described the structure of NETs (on the basis of electron microscope images) as thin, smooth strands of DNA with diameters ranging from 15–17 nm and globular domains approximately 25 nm in diameter. Additionally, the presence of histone proteins including H1, H2A, H2B, H3, and H4 as well as of the H2A-H2B-DNA complex within the globular domains of neutrophil traps has also been shown [28]. The trapping of pathogenic microbes within a net composed of DNA fibers prevents their spread and allows the concentration of antimicrobial factors at the site of the infection [29].

Analysis of the structure of NETs has demonstrated the presence of proteins found in neutrophil granules such as neutrophil elastase (NE), cathepsin G, myeloperoxidase (MPO), proteinase 3, bactericidal permeability-increasing factor (BPI), lactoferrin, cathelicidin hCAP/LL37, pentraxin 3, lysozyme, or α-defensin [30].

Enzymes that are key to the formation of NETs include:

- NADPH oxidase engaged in the process of ROS production;
- NE degrading intracellular proteins and initiating the disintegration of the cell’s nucleus;
- Protein arginine deiminase 4 (PAD4), which citrullinates histones to facilitate decondensation and release of chromosomal DNA;
- Gasdermin D, which is responsible for the generation of pores in the cell’s membrane allowing the expulsion of traps beyond the cell wall.

Although NETs play a beneficial role in the defense of their host from pathogens, extended inflammation connected with their presence may cause a cascade of unfavorable reactions. The example of such reactions is the production of antibodies against the host’s DNA (autoimmunization), damage to surrounding tissue, or the occurrence of atherothrombotic events [3,4,7,20,30,31,32,33,34,35,36,37].

Research into the mechanisms regulating the generation of NETs has shown that, among inflammatory cytokines engaged in the immunopathogenesis of COVID-19 is IL-1β. It is the key inductor in the creation of NETs, both under in vivo as well as in vitro conditions. An opposite situation has also been observed, where it is the NETs that stimulate macrophages to increase the production of the IL-1β precursor, which indicates a certain positive link between IL-1β and NETs. This prompted the formulation of a hypothesis that a coupled loop created by IL-1β and NETs may lead to excessive damage of the alveoli and pulmonary endothelium observed in patients with severe progression of COVID-19 [3,5,38,39,40]. Damaging the endothelium causes the release of the von Willebrand factor (vWF), which activates blood platelets and neutrophils. Activated platelets additionally stimulate neutrophils to produce NETs, which become a structure upon which blood platelets, erythrocytes, and fibrines aggregate aiding the development of clots [38,41,42,43,44].

Excessive formation of NETs in COVID-19 patients is confirmed by elevated concentrations of NETs markers such as circulating free DNA (cfDNA) or the levels of DNA-MPO and DNA-NE complexes. Intensified citrullination of H3 histones (citH3) has also been described in COVID-19 patients. Both citH3 as well as cfDNA correlated positively with the number of leukocytes and neutrophils, while cfDNA correlated positively with CRP and LDH. Activity of NE in blood was over 30 to 60 times higher, respectively, to those with the severe or mild progression of the disease, in COVID-19 sufferers when compared to that of healthy subjects [13,45]. It has also been shown that sera obtained from COVID-19 patients are strong stimulators of NETs formation in control neutrophils [45]. Schönrich et al. (2020) cite studies of Imai et al. (2008) who have shown the reduction in inflammation and increased survivability of mice infected with SARS-CoV after the inhibition of the NF-κB signaling pathway [5,46].

**Figure 1:**
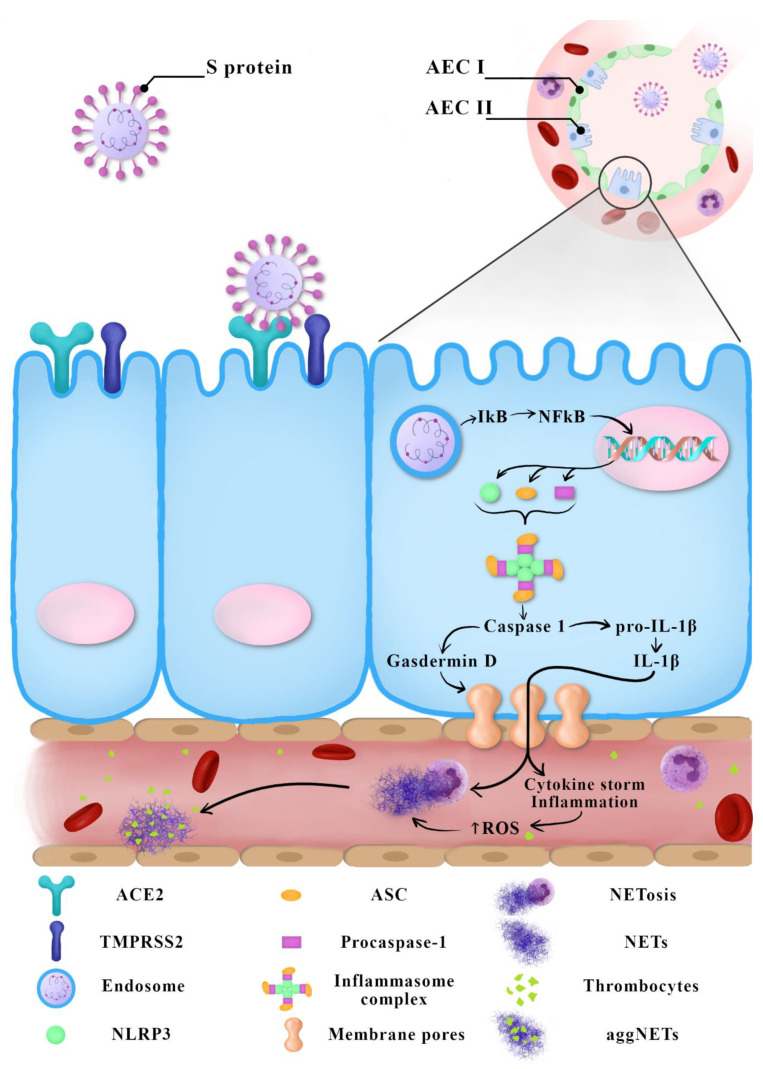
Suggested COVID-19 immunopathogenesis—the latest reports.

SARS-CoV triggers a significantly stronger innate response induced through the NF-κB pathway in older experimental animals than in younger ones. This may explain a more severe progression of COVID-19 in older people. During a SARS-CoV-2 infection, the level of ROS in older people may cause the excessive activation of NF-κB, which results in inflammation-caused tissue damage [33]. Similarly, the greater susceptibility of men to oxidative stress may be a cause of their greater susceptibility to having a more severe progression of COVID-19 than women [5]. Figure 1 summarizes the current knowledge regarding possible COVID-19 immunopathogenesis.

## 4. NETs and COVID-19 Progression in People with Concurrent Diseases

It is estimated that over 1.7 billion people (approximately 20% of the world’s population) fall into the increased risk of severe COVID-19 group because of the coexistence of cardiovascular and metabolic (mainly diabetes) diseases [5].

Severe cases of COVID-19 often co-occur with acute respiratory distress syndrome (ARDS), chronic inflammatory states, or even sepsis. In such instances, intensification of procoagulatory activity, disseminated intravascular coagulation (DIC), and endothelial damage may also occur with available literature indicating elevated values for inflammation markers including a rise in the number of neutrophils, high concentrations of CRP, LDH, fibrinogen as well as IL-1, IL-6, IL-8 [23,47].

Cardiovascular diseases including coronary heart disease, arrhythmia, or hypertension have an overall higher mortality rate in connection with COVID-19. ACE2 induces vasodilatory activity through Ang-(1–7) and the Mas receptor. This is the reason a decrease in ACE2 activity connected with the penetration of the cell by SARS-CoV-2 causes vessel contraction leading to hypertension [41,43,48,49,50]. The later infiltration of neutrophils and their degranulation and the release of NETs contributes to excessive inflammatory response and progression of diseases affecting the circulatory system [51,52]. As described by scientific literature, excessive generation of NETs results in procoagulatory properties, and SARS-CoV-2 infection is associated with hypercoagulability, which predisposes to venous thromboembolism (VTE) [29,53]. In addition, NETs initiate both the extrinsic (by presentation of tissue factor-TF) and contact (by augmenting activation of factor XII-FXII) pathways of blood coagulation, as well as trapping and activating platelets [54]. An important element of NETs, neutrophil elastase, impacts the inactivation of the tissue factor pathway inhibitor (TFPI). Ammollo et al. (2011) have proven that the directly connected to NETs surplus of extracellular histones may inhibit the activation of the anticoagulatory protein C influencing the activity of the epithelial cofactor thrombomodulin (TM), which results in disturbances in the inactivation of thrombin playing an active part in the conversion of fibrinogen to insoluble fibrin and, thus, to the formation of blood clots [55,56]. Price et al. (2020) present a hypothetical model of pulmonary embolism and pulmonary microcirculation thrombosis in the course of COVID-19, taking into account the share of NETs [41]. High levels of cfDNA and histones, which can activate thrombin production, were observed during a SARS-CoV-2 infection [3]. Moreover, NETs were localized in lung vessels during autopsy specimens of COVID-19 patients [45]. Thrombosis and thromboembolism (TE) associated with COVID-19 are different from sepsis-associated disseminated intravascular coagulation. However, high blood levels of D-dimers produced to dissolve clots may be an indicator for severe progression of COVID-19 [5,57]. Moreover, patients previously diagnosed with a thrombotic event showed significantly higher levels of calprotectin, a neutrophil activation marker [58]. A review and meta-analysis of the available literature on thromboembolism cases in COVID-19 patients unanimously indicates a high level of TE rates in these patients and a correlation with a high risk of death [59]. Jacqui Wise quotes Beverley Hunt (medical director of Thrombosis UK) “Thrombosis is definitely contributing to the high mortality rate from covid” and also mentions the lack of explicit recommendations for therapeutic management of thrombosis in COVID-19 patients, which is a serious medical problem [60]. The results of studies by other authors unequivocally confirm that hypercoagulability and thrombotic events are driven by NETosis, contact pathways of blood coagulation activation and complement, and their repeatedly amplified feedback loops. Researchers are proposing C5a blockers, plasma kallikrein and FXIa inhibitors, and NETs-derived histone neutralizing agents as new therapeutic directions [61].

It has been proven that COVID-19 has been associated with thromboembolic episodes not only during the period of infection but even in the convalescence period. Fan et al. (2020) describe cases of delayed thromboembolic complications in patients after COVID-19. The authors suggest that low-grade inflammation associated with endothelial activation status may persist following SARS-CoV-2 infection, posing a risk of thrombotic events [62,63]. Sawadogo et al. (2020) points out that if research confirms NETs as the leading cause of the severe and fatal course of COVID-19, perhaps we should prepare to define a new non-communicable inflammatory disease among convalescents—“Post-COVID-19 Syndrome”. The authors suggest the need to monitor NETs biomarkers, but also to extend the research to the evaluation of autoimmune markers: ANCA, anti-cyclic citrullinated peptide, rheumatoid factor, and anti-NET antibodies (ANETA) [64].

In cases with concurrent diseases, increased oxidative stress, which predisposes patients to severe COVID-19, is of key importance. Hyperglycemia and hypoxia observed during problematic ventilation of patients with diabetes and undergoing a SARS-CoV-2 infection promote the production of ROS, which may cause NLRP3-mediated pyroptosis [5]. Hyperglycemia stimulates neutrophils to release NETs, which can intensify the “cytokine storm” often leading to systemic inflammatory response syndrome (SIRS) or sepsis within the course of COVID-19. In response to hyperglycemia, neutrophils produce calcium-binding S100 A8/A9 proteins (S100A8/A9), which stimulate the production of IL-6 and thrombopoietin, possibly leading to the formation of micro-clots during COVID-19 [7,45]. It must be stressed that greater expression of ACE2, a cause of greater susceptibility to SARS-CoV-2 infection, has been seen [7].

## 5. Prospective COVID-19 Therapies Based on the Regulation of NETs Formation

The year 2020 has become a period of intense research into ways to develop appropriate procedures for the treatment of patients diagnosed with COVID-19. Due to serious threat to patients’ lives, it is necessary to gain a thorough understanding of the immunopathogenesis of this disease as well as to identify the largest number of strategic grasping points of personalized therapies, thus increasing the chances for the successful treatment of every patient.

Studies into the development of drugs meant to regulate NETs production are especially interesting. Potential medications include inhibitors of particles that are essential for NETs creation such as NE, PAD4, and gasdermin D as well as substances allowing the dissolution of the excessive amount of NETs. It has been shown that Cl-amidine (a PAD4 inhibitor) may limit the creation of NETs and, therefore, prevent the development of thrombosis; however, so-far, these observations have only been carried out on animal models [47,65,66]. Other factors that inhibit NETs generation, gasdermin D inhibitors, are currently at the pre-clinical trial phase. There are reports that disulfiram, a drug used to treat alcoholism, effectively suppresses gasdermin D and reduces lung damage in animal models [3].

Additionally, there are declarations that dipyridamole (adenosine uptake inhibitor), an FDA approved antiaggregatory drug, is a NETs formation inhibitor (through the activation of adenosine A2A receptors) [16], a fact that has recently been reported by Zuo et al. [45].

There is also information that dornase alpha or the recombinant human deoxyribonuclease (DNase1) administered by inhalation may be used to dissolve NETs in the respiratory pathways of patients with cystic fibrosis to alleviate symptoms connected with inflammation. It is usually delivered using a nebulizer, but due to the high risk of aerosolizing the SARS-CoV-2 virus and creating a threat to health care workers and other patients, its use is avoided [3]. The utilization of DNase1 may also, as a side effect, cause the release of proteases present within the structure of NETs such as NE, which may have potential cytotoxic properties [67]. Clinical studies into the identification of NE inhibitors are currently very advanced. Sivelastat, a NE inhibitor, has been approved for the treatment of ARDS in Japan and South Korea. New generation NE inhibitors including lonodelestat (POL6014), alvelestat, CHF6333, and elafin have completed phase one of testing [3,38,68].

It is assumed that anakinra, canakinumab, and rilonacept, IL-1β inhibitors, may disrupt the IL-1β/NETs feedback loop. Studies meant to verify the effectiveness of administering anakinra in COVID-19 are currently in progress [3,39,68].

There is also research into colchicine, which may suppress both the recruitment of neutrophils to inflammation sites as well as inhibit the generation of IL-1β [17,67].

Since the cells of the endothelium express ACE2 and are susceptible to SARS-CoV-2 infections, there is a chance to check the spread of the infection using soluble particles of ACE2, which would probably also inhibit the recruitment of neutrophils and the excessive formation of NETs.

Due to the above-mentioned possible interactions between neutrophils and thrombocytes, it is worth pointing out that the administering of aspirin reduces the formation of neutrophil traps in pulmonary microcirculation and blood plasma [67].

It has been observed that glyburide, a drug for the treatment of diabetes classified as a sulfonylurea, may block the activation of the NLRP3 inflammasome through the inhibition of ATP-sensitive K+ channels. It is, however, assumed that the dose necessary to attain the desired effect in vivo would be too large and would most likely cause hypoglycemia [38]. Metformin (*N*,*N*-dimethylbiguanide)—another antidiabetic drug—directly binds alarmin HMGB1, suppressing its proinflammatory properties and, indirectly, may contribute to the inhibition of NETs overproduction [19,67,69,70]. The proposed therapeutic strategies in the course of COVID-19 based on the inhibition of NETs formation and anti-inflammatory effects are summarized in Table 1.

## 6. Summary

The present work clearly indicates the significant participation of NETs formation in the immunopathology of COVID-19 and the connected-with-it severe complications resulting from the intensification of the process of inflammation that is key to the course of a SARS-CoV-2 infection. The contribution of neutrophils and NETs, along with other immune system cells and their transmitters to the immunological response accompanying COVID-19, is still in need of extraordinarily precise and extensive studies. However, the results available at this stage of research allow the identification of the regulation of NETs and their markers as objects of new, dedicated therapeutic strategies meant to increase the chances for survival and the improvement of the disease severity of COVID-19 and/or mortality rates. The continuation of research on the role of NETosis in COVID-19 seems to be a priority over the scientific evidence gathered so far. Many threads need to be clarified, for example the assessment of the importance of other receptors involved in the antiviral response (for example, the TLR family: TLR3, TLR7, or TLR9). Understanding the mechanisms of regulating the NETs formation phenomenon may be used in adjunctive therapy in the course of COVID-19. Moreover, determination of NETs markers may be useful not only in predicting the severity of the course of COVID-19 but also as a prognostic indicator of “Post-COVID-19 Syndrome”.

## Figures and Tables

**Table 1:** Prospects of COVID-19 therapies based on extracellular neutrophil traps (NETs) blocking and anti-inflammatory action.

	Treatment	Target	Action
Inhibition of NETs formation	NE inhibitors: - Sivelestat - Lonodelestat - Alvelestat - Alafina - CHF6333	NE	Antiprotease
	PAD4 inhibitor: - Cl-amidine	PAD4	Inhibition of histone citrullination
	Gasdermin D inhibitor: - Disulfiram	Gasdermin D	Inhibition of pore generation in the cell membrane
	Dipyridamole	A2A adenosine receptors	Adenosine uptake inhibitor
	Aspirin	NF-κB p65 signaling pathway	Decrease in NETs formation
NETs dissolution induction	Dornase alfa	DNA	DNA degradation
Blocking IL-1β activity	IL-1β inhibitors: - Anakinra - Canakinumab - Rilonacept	IL-1β	Disrupting the feedback loop IL-1β/NETs
	Colchicine	- Neutrophils - IL-1β	- Inhibition of neutrophil recruitment - Blocking the secretion of IL-1β
Anti-inflammatory effect	Glyburide	ATP-sensitive K+ channels	Blocking the activation of the NLRP3 inflammasome
	Metformin	Alarmin HMGB1	Suppressing proinflammatory properties

## Data Availability

Not applicable.
